# Fabrication of inverted zinc oxide photonic crystal using sol–gel solution by spin coating method

**DOI:** 10.1186/1556-276X-8-306

**Published:** 2013-07-02

**Authors:** Kuo-Min Huang, Chong-Lung Ho, Heng-Jui Chang, Meng-Chyi Wu

**Affiliations:** 1 Institute of Electronics Engineering, National Tsing-Hua University, Hsinchu, 300, Taiwan

**Keywords:** Inverted ZnO, Photonic crystal structure, Sol–gel solution, Near-ultraviolet

## Abstract

Inverted zinc oxide photonic crystal structures were fabricated from polystyrene sphere (PSS) template using the sol–gel solution of ZnO by spin-coating method. It is easily able to control and fabricate the photonic crystal structures using the self-organized PSS with a size of 193 nm. The inverted ZnO photonic crystal structures observed show the (111) tendency of the hexagonal compact arrangement formation. The resulting structures possess the photonic band gaps in the near-ultraviolet range and exhibit an enhanced photoluminescence spectrum. The technology can effectively increase the light output intensity or efficiency for the applications of optoelectronic devices.

## Background

Since photonic crystals (PhCs) were first proposed in 1987 by Yablonovitch [1] and John [2], they have been studied with great interest as a means of localizing light and modifying the emission properties of embedded light sources [3]. Material infiltration of three-dimensional (3D) polystyrene sphere (PSS) PhC has been a versatile method to fabricate the so-called inverted structure, which has long-range order, high filling fraction, and refractive index contrast required to exhibit a photonic band gap. Infiltration has been recently achieved by various methods, including chemical bath deposition [4], electrodeposition [5], and low-pressure chemical vapor deposition [6]. To achieve both high filling fractions and good luminescence properties of this material has been proven difficult [7]. In spite of the few studies regarding the sol–gel method, this method has some advantages, such as the easy control of chemical components and fabrication of thin film at low cost to investigate the structural and optical properties of ZnO thin films. Several groups have, therefore, studied the emission properties of lasing dyes or quantum dots infiltrated into inverted opal backbones [8]. Teh et al. reported that the optical gain of the 3D ZnO inverse opal fabricated by electrodeposition is further enhanced due to the localized defect modes within the primary photonic pseudogap. Teh et al. reported the room-temperature ultraviolet lasing and the mechanisms of lasing modes in 3D ZnO inverse opals fabricated via colloidal templating with electrochemical infiltration. They further investigated the mechanisms of lasing modes and deduced that periodic structures would facilitate strain-induced change in lasing energy and provide modulation in refractive index for enhanced light confinement as well as optical feedback. They concluded that the periodic photonic structure plays a role, i.e., the modulation in refractive index would enhance the light confinement as well as the optical feedback [9]. The inverted ZnO PhC possesses a wide electronic band gap (3.2 eV at room temperature) and high exciton binding energy (60 meV), which makes it an efficient short-wavelength light source in the near ultra-violet (NUV) spectrum. Its refractive index (2.26) is too low to produce a full (i.e., omnidirectional) photonic band gap but sufficient for the formation of directional pseudogaps. In this article, we report the fabrication of inverted ZnO PhC using sol–gel solution by spin coating method and demonstrate the morphology, reflection spectra, and luminescence in the NUV region for the examination of the process on inverted ZnO PhCs.

## Results

**Figure 1:**
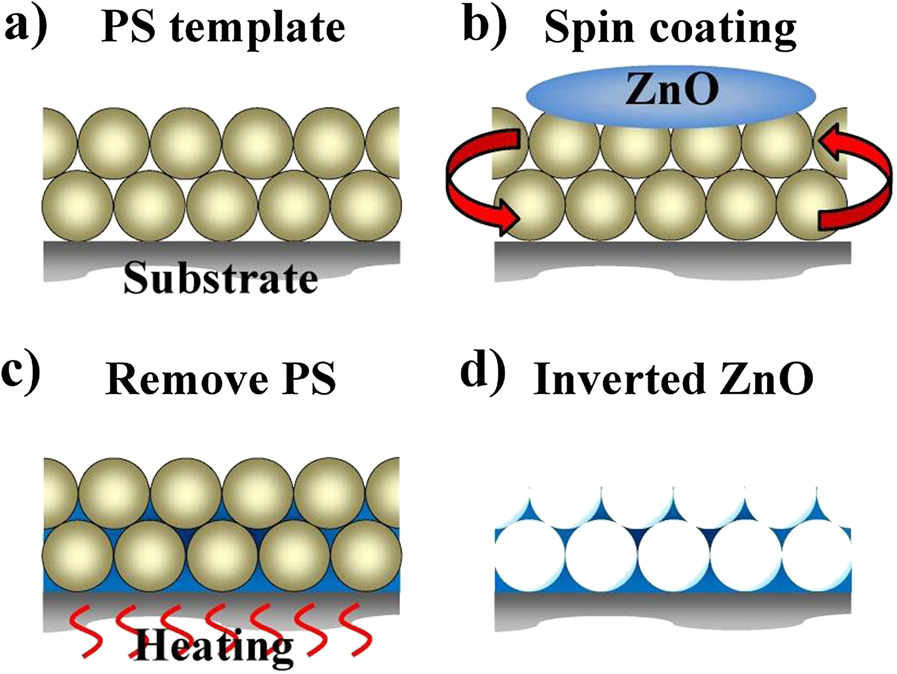
**Schematic fabrication process of the inverted ZnO PhC structure using the sol–gel solution. (a)** PSS template, **(b)** spin coating, **(c)** removal of the PSS under a thermal treatment, and **(d)** inverted ZnO PhC structures.

**Figure 2:**
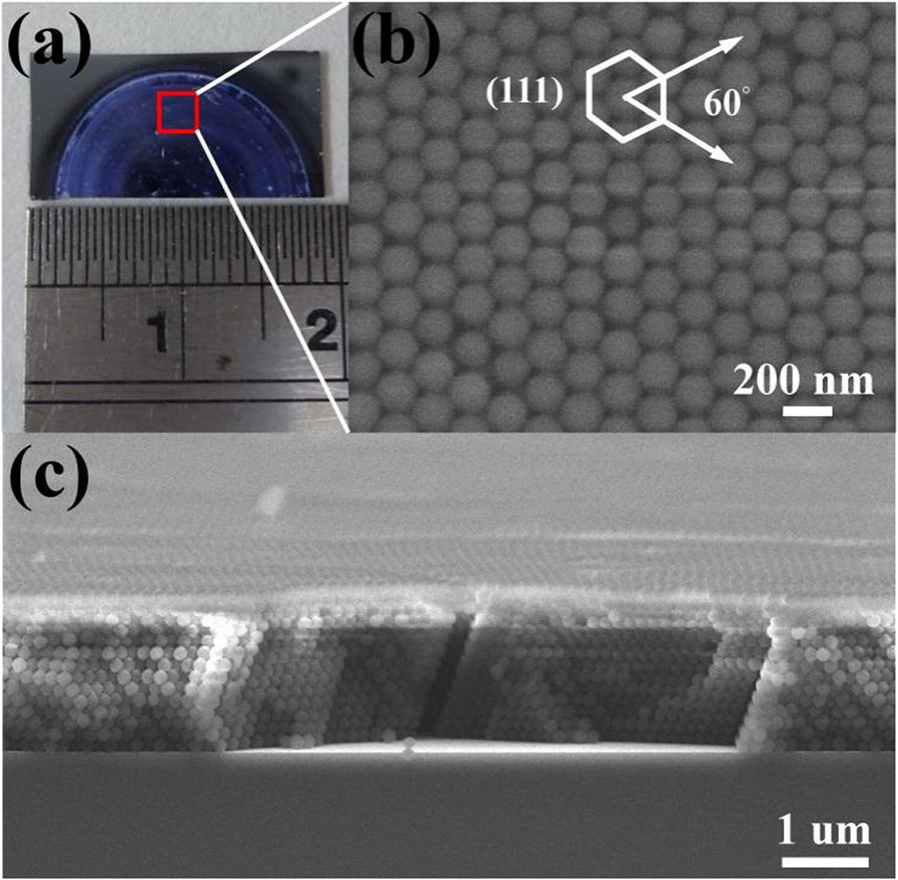
**Optical and FE-SEM images. (a)** Optical image of the self-assembled periodic arrangement polystyrene spheres formed on silicon substrate. **(b)** Top-view and **(c)** cross-section magnification FE-SEM images of the self-assembled multilayer of polystyrene spheres.

**Figure 3:**
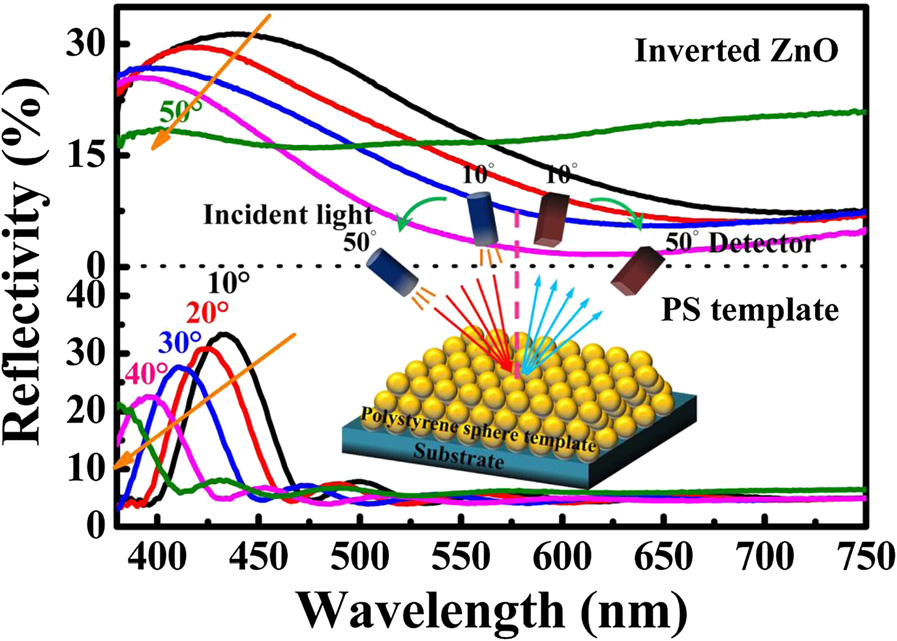
**Reflection spectra of PSS PhC templates and inverted ZnO PhC measured in (111) direction.** Incident angles are 10°, 20°, 30°, 40°, and 50°. The inset presents the measured conditions in this study.

**Figure 4:**
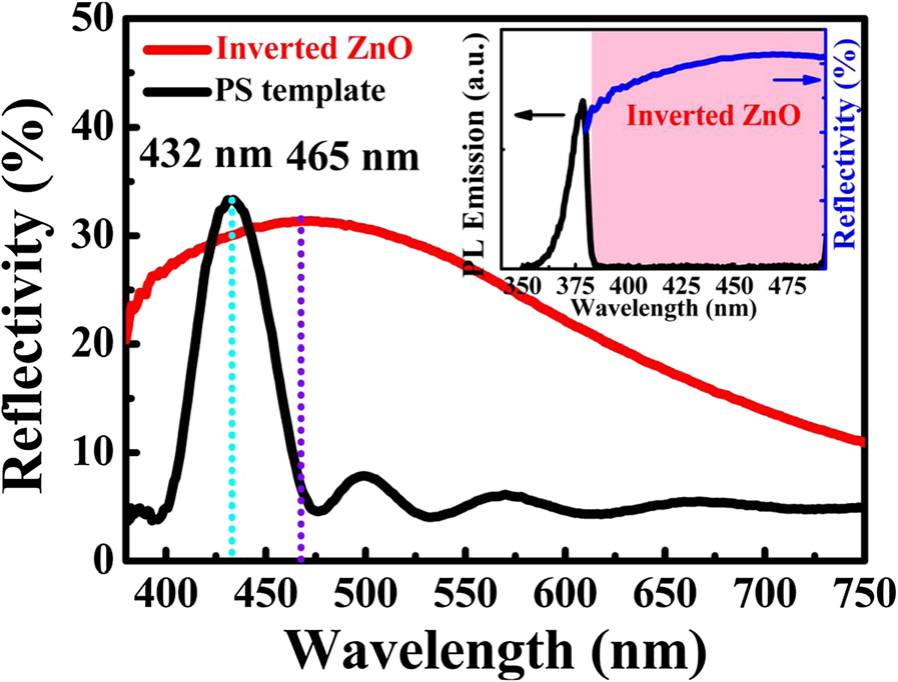
**Reflection spectra of the structures.** PSS PhC template (black curve) and inverted ZnO PhC (red solid curve) structures. The inset shows the PL emission and reflectivity of the inverted ZnO PhC. The blue and violet broken lines are the locations of peaks.

**Figure 5:**
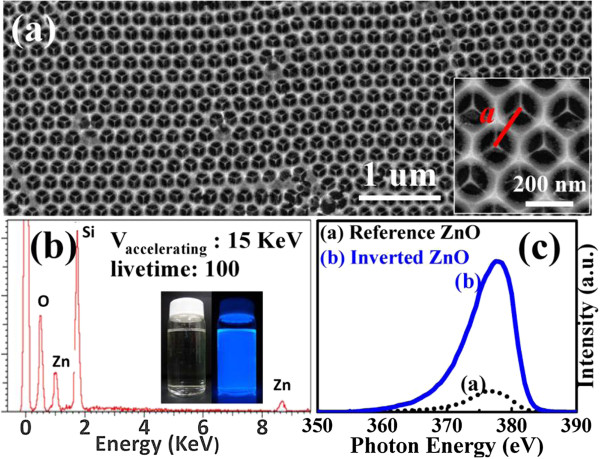
**FE-SEM image, EDS spectrum, and comparison of Pl spectra. (a)** Top view FE-SEM image of low magnification of the inverted ZnO PhC structure. The inset displays the high magnification of the FE-SEM image, showing the honeycomb-like structure produced by spin coating method. **(b)** EDS spectrum recorded from the inverted ZnO PhC structure. **(c)** Comparison of the exciton emission intensity of the PL spectra for the reference ZnO (black short dot curve) and the inverted ZnO PhC structure (blue solid curve) under the same excitation condition.

Inverted ZnO structures were fabricated using PSS suspension with diameters of 193% ± 5% nm. The PSS suspension was dispersed in aqueous solution. The volume fraction of the solution is around 2.0 wt% PSS which was assembled onto the silicon and glass substrates by horizontal self-assembly method. The thickness and size of substrate are about 350 μm and 20 mm × 20 mm, respectively. Prior to spreading, the solution underwent hydrophilic treatment using ultraviolet ozone plasma about 15 min in order to easily cover the substrate. The PSS suspension on the cleaned substrate was kept in glass covers onto the hot plate at 30°C for about 1 h. Figure 1 illustrates the schematic fabrication process of the inverted ZnO PhC structure using the sol–gel ZnO by spin coating method to deposit the sol–gel solution with dihydrate zinc acetate, monoethanolamine, and isopropyl alcohol. The used temperature for the ZnO synthesis is 60°C with stirring time of 90 min. The drying process of the spread suspension can be observed from the central region of the sample as water evaporated from the aqueous colloidal solution and sequentially organized the PSS, as shown in Figure 1a. ZnO nanoparticles were prepared by spin coating method to deposit the sol–gel solution with dihydrate zinc acetate and monoethanolamine. After the drying process, the PhC structures of the PSS were formed on the substrate. The mixing concentration and temperature of ZnO synthesis were 0.1 M and 75°C, respectively, with the stirring time of 60 min, keeping the solution stable for spin coating after 24 h. Figure 1b displays the sol–gel solution of the ZnO drop on the PSS template to spin it. Inverted ZnO PhC structures integrated with ZnO nanoparticles were formed by removing the PSS under a thermal treatment of 400°C for 1 h, as shown in Figure 1c,d. Further analyses of inverted ZnO structures were characterized using photoluminescence (PL) and field-emission scanning electron microscopy (FE-SEM; JEOL 6500 F, Tokyo, Japan). The crystalline quality of the PSS template is among the most important parameters in determining the performance of inverted ZnO PhC in optical applications. The formation of point defects can have an enormous impact on the reflection properties. Figure 2a shows an image of the periodic arrangement of PSS structures with a diameter range of 15 mm formed on the substrate by the horizontal self-assembly method. The structures appear blue iridescence. The detailed organization of the spheres is investigated by FE-SEM. Figure 2b is a top-view magnification of the FE-SEM image, which shows a relatively well-organized arrangement of the ordered close-packed face-centered cubic (fcc) structure along the (111) planes. The ordering is reasonably good, although point defects are observed in some areas, which may be produced by a variation in sphere size. A closer examination presented in Figure 2b shows perfectly ordered arrangement. The cross-section image of a larger magnification is tilted with an angle of 10°, as shown in Figure 2c. It was observed that the spheres were also organized as ordered close-packed fcc structure with the (111) planes parallel to the substrate surface. Optical characterization of the PhC could give the position of the stop band and its angle-dependent behavior. For optical characterization, reflectivity is recorded from the (111) plane of the crystals. Figure 3 shows the reflection spectra of the PSS PhC templates and inverted ZnO PhC measured in (111) direction at the incident angles of 10°, 20°, 30°, 40°, and 50°. The inset presents the measured conditions in this study. An inspection of this figure reveals that the spectrum of PSS PhC templates measured at the incident angle of 10° exhibits a maximum reflection of 34% at the wavelength of 432 nm. The calculated wavelength of the reflection peak is 432 nm according to the modified Bragg's law [10] by considering the colloidal-sphere diameter to be 193 nm. The reflectivity of the inverted ZnO PhC can correspond to the Bragg reflection from the ordered porous structures. The reflectivity of the inverted ZnO PhC can still be identified using the angle-dependent phenomenon. The reflectivity peak of the inverted ZnO PhC shifts with increasing incident angle towards high energy band. Maybe the broadband reflectivity is caused by the non-stoichiometry of this inverted ZnO PhC. When the angle of incident light increases, the reflection spectral peak shifts towards the short wavelength range. The shift of the reflection spectrum with increasing angle of incident light indicates the pseudo-band gap nature of the PhC. Fabry-Perot (F-P) oscillations are observed on both sides of the reflection maximum. The estimated thickness of the PSS PhC from the F-P oscillation is 2 μm, with 13 numbers of periodic arrangement layers [11]. The reflection spectra of the PSS PhC template and inverted ZnO PhC structures are shown in Figure 4. The spectral position of the reflection maximum *λ* = 432 nm (in Figure 4) in the PSS PhC template corresponds to the Bragg condition *λ* = 2*dn*_eff_ with the effective value of refraction index, *n*_eff_ = 1.37, in fair agreement with the calculation from the following Equation (1). In our case of the fcc lattice, the plane-to-plane distance is *d* = (2/3)^1/2^*D*_PS_ along the <111 > direction, where *D*_PS_ = 193 nm and *D*_inverted__ZnO_ = 200 nm are the diameters of the PS spheres and inverted ZnO PhC structure, respectively. In the general case of the three-component system, *n*_eff_ is governed by the relation [12]

(1) $$n_{\mathrm{eff}}^{2}=n_{1}^{2}f_{1}+n_{2}^{2}f_{2}+n_{3}^{2}f_{3},$$

where *n*_1_ = 1.48, *n*_2_ = 2.0, *n*_3_ = 1; *f*_1_, *f*_2_, and *f*_3_ are the refraction indices and volume proportions of PSS, ZnO, and air, respectively (*f*_1_ + *f*_2_ + *f*_3_ = 1). It should be taken into account that for the volumetric proportion of PSS, *f*_1_ = 0.67, the porosity being *f*_3_ = 0.33 (*f*_2_ = 0) as contrasted from the inverted ZnO PhC structure, where *f*_2_ = 0.42 and *f*_3_ = 0.58 (removed PSS, *f*_1_ = 0) [12]. From Equation (1), calculate the filling fraction. The calculated effective index of refraction of the inverted ZnO PhC structure is *n*_eff_ = 1.42. The reflection maximum of such a structure ought to be at 465 nm for *D*_inverted__ZnO_ = 200 nm. This is in good agreement with the experimental value of 465 nm (red solid curve in Figure 4). The inverted structure is partly filled with ZnO. In the weight gain, the infiltration makes up 25% of the calculated value because the pores are being completely filled. In this study, the range of photonic stop band overlaps with the visible band of the inverted ZnO PhC. The effect of the stop band is not observed on the visible band because this structure has only 20% of reflectance in this wavelength range. The observed result of the inverted ZnO PhC is the enhanced light confinement when its primary pseudogap approaches the ZnO emission [9]. SEM images recorded from the inverted ZnO structure are depicted in Figure 5a which shows a top view image of low magnification of the inverted ZnO PhC. An inspection of the inset of Figure 5a reveals that the honeycomb-like arrangement of the ZnO nanoparticles is integrated during the growth process, where *a* is the lattice constant of the primitive cell. It means that the center of any inverted ZnO is close to the next one. In addition, the uniformity of ZnO PhCs can reach a micrometer scale. The composition is confirmed to be ZnO nanoparticles, analyzed through energy dispersive X-ray spectroscopy (EDS), as shown in Figure 5b, where the silicon signature is from the silicon substrate. PL spectra were attained from the inverted ZnO PhC to disclose their collective optical properties. The inset images are the sol–gel solutions of the ZnO nanoparticles exposed to the UV light of 365 nm, showing blue fluorescent, and those not exposed to the UV light. The PL measurements were performed at room temperature using a 325-nm He-Cd laser as the excitation light source. As shown in Figure 5c, a strong NUV emission (curve a) at 378 nm is observed for the ZnO reference sample, and the emission (curve b) for the inverted ZnO PhC is attributed to the near-band-edge emission due to the exciton-related activity [13]. The emission peak is related with the free exciton recombination in ZnO at room temperature and has the FWHM of 8 nm (65 meV) for the inverted ZnO PhC. Surprisingly, although the volume fraction of ZnO nanocrystals in the inverted structure is only one-fifth of that in the reference sample, the NUV emission of the inverted ZnO PhC reveals a higher intensity than that of the reference sample. There is no distinct difference in chemical environment between the inverted ZnO PhC and the reference sample, which indicates that the marked enhancement of PL intensity refers to the effect of 3D ordered porous structure. Considering that the walls of the inverted structure are sandwiched by air, a ZnO porous structure could be regarded as a semiconductor-insulator nanostructure, in which the semiconductor is surrounded by the insulator with a smaller dielectric constant than the semiconductor material. Such a structure should induce an increase in oscillator strength and exciton binding energy due to the dielectric-confinement effect [14,15]. This effect could improve the exciton properties of semiconductor with larger sizes than the quantum-confinement effect due to the long-range nature of the Coulomb interaction [15]. Therefore, a more intensive exciton emission is expected from the inverted ZnO PhC due to the dielectric confinement effect. It is, thus, suggested that the dielectric confinement effect is one of the possible factors concerning the PL enhancement of the inverted ZnO PhC. Structure disorder is also one of the possible factors concerning this phenomenon [16]. The unintentional disorder in the inverted ZnO PhC could cause intense light scattering and could increase the absorption efficiency of the excitation light, which helps obtain a high luminescence intensity. It has been previously demonstrated that intense scattering induces a remarkable PL enhancement in ZnO-SiO_2_ composite opals [17]. Another possible factor causing the emission enhancement may be an improvement in the luminescence extraction efficiency due to the textured top surfaces of the inverted ZnO PhC [13].

## Summary and conclusions

We have successfully fabricated the inverted ZnO PhC structure using the sol–gel solution of ZnO by spin coating method. Sol–gel is capable of producing high filling fraction inverted opal materials with very good crystalline quality. The results of the inverted ZnO structure exhibit clear strong NUV photoluminescence at the wavelength of 378 nm, which makes them interesting candidates for studying the characteristics of modified spontaneous and stimulated emission in active ZnO PhCs. The combination of aqueous chemical growth and nanosphere lithography is expected to provide a facile, large-scale, and low-cost fabrication method at low temperatures, which shall be of significant value for practical applications of the grown PhCs.

## Competing interests

The authors declare that they have no competing interests.

## Authors’ contributions

KH and HC carried out the design and the experiment. CH set up the measurement system. MW conceived of the study and facilitated its coordination. All authors read and approved the final manuscript.
